# Optimizing Voice Outcomes After Recurrent Laryngeal Nerve Transection: The Role of Early and Supplemental Injection Laryngoplasty

**DOI:** 10.1002/kjm2.70185

**Published:** 2026-03-09

**Authors:** Luo‐Wei Chan, Hsin‐Yi Tseng, Che‐Wei Wu, Tzu‐Yen Huang

**Affiliations:** ^1^ School of Medicine, College of Medicine Kaohsiung Medical University Kaohsiung Taiwan; ^2^ Department of Otorhinolaryngology‐Head and Neck Surgery, Kaohsiung Medical University Hospital Kaohsiung Medical University Kaohsiung Taiwan; ^3^ Department of Otorhinolaryngology, School of Post‐Baccalaureate Medicine and School of Medicine, College of Medicine Kaohsiung Medical University Kaohsiung Taiwan; ^4^ Department of Otolaryngology‐Head and Neck Surgery, Kaohsiung Medical University Gangshan Hospital Kaohsiung Medical University Kaohsiung Taiwan

**Keywords:** early and supplemental injection, hyaluronic acid, injection laryngoplasty, recurrent laryngeal nerve, voice outcome

1

Vocal fold paralysis (VFP) caused by recurrent laryngeal nerve (RLN) transection can lead to persistent glottic insufficiency, progressive vocal fold atrophy, and maladaptive compensatory phonatory behaviors if early intervention is not provided. Injection laryngoplasty (IL) is a minimally invasive treatment option for unilateral VFP. Early IL within 3 months has been supported by some reports [1, 2], although the limited duration of benefit in certain patients has been considered a drawback [3]. Here, we present a case to illustrate treatment considerations in this context.

A 59‐year‐old man presented with a 3‐month palpable neck mass and two weeks of hoarseness. He had undergone bilateral thyroidectomy for papillary thyroid carcinoma (PTC) 20 years earlier. Imaging suggested recurrent left PTC (Figure 1A), and preoperative laryngoscopy revealed delayed left vocal fold movement. Revision thyroidectomy with central neck dissection was performed. Intraoperatively, tumor invasion of the left RLN required nerve transection and immediate reinnervation (Figure 1B). Pathology confirmed PTC recurrence.

**FIGURE 1:**
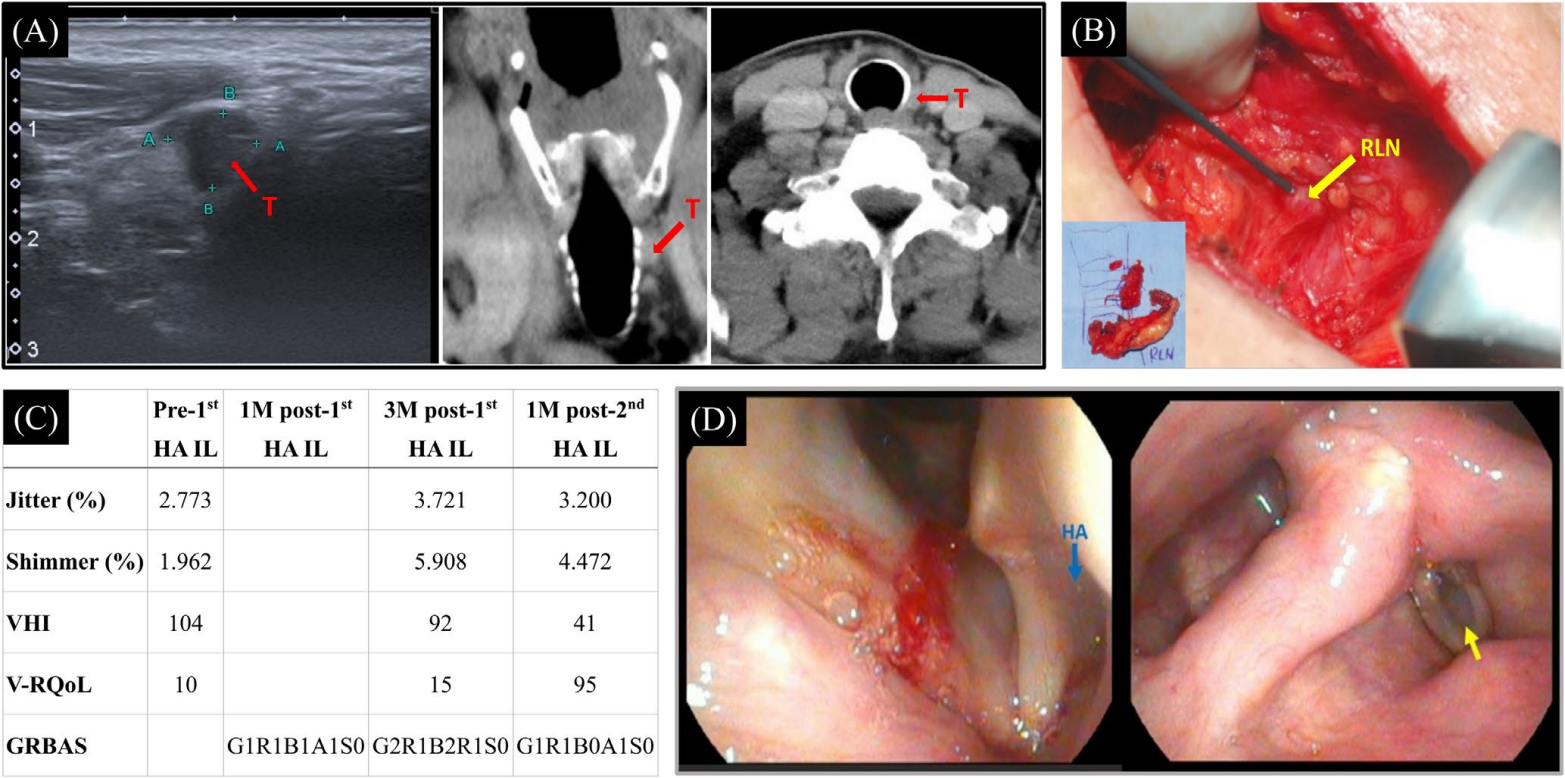
(A) Preoperative image—A heterogenous thyroid tumor (T) was noted on neck ultrasonography and computed tomography. Tumor invasion to the left tracheoesophageal groove was demonstrated. (B) Intraoperative findings—the left recurrent laryngeal nerve (RLN) was invaded by the tumor. (C) Objective and subjective voice outcomes of each hyaluronic acid (HA) injection laryngoplasty (IL), including jitter, shimmer, voice handicap index (VHI), voice‐related quality of life (V‐RQOL), and GRBAS scores. (D) Pre‐supplemental laryngofiberscope—performed at 3 months after the first injection still showed HA (blue arrow), but a glottic gap (yellow arrow) was noted when phonation. Therefore, a supplemental HA IL is then performed.

Postoperatively, the patient reported worsening hoarseness, choking, and globus sensation, with deterioration in objective and subjective voice measures (Figure 1C). Early hyaluronic acid (HA) IL using a commercially available HA‐based filler (Restylane Lyft with lidocaine; Galderma, Switzerland) with an injection volume of 0.9 mL targeting the vocalis muscle was performed 2 weeks after surgery due to persistent symptoms and a low likelihood of short‐term spontaneous recovery from clinically significant glottic insufficiency. One month later, he reported satisfactory improvement and discontinued speech therapy due to work abroad. Three months after IL, however, both objective and subjective voice measures worsened. Despite the presence of HA on laryngofiberscopy, a renewed glottic gap was observed (Figure 1D), and supplemental IL was performed 5 months after the initial injection because of symptomatic glottic insufficiency despite persistence of the injected material, using the same HA product, with 0.8 mL injected into the left vocalis muscle, targeting the same functional region with a slightly more medial adjustment. One month later, subjective parameters improved markedly and objective parameters improved mildly, with stable voice quality thereafter for 1 year. Following the supplemental injection and oncologic treatment, the patient has not required further injection laryngoplasty or additional cancer‐related interventions to date.

We support an “early injection” strategy to restore basic vibratory function during the period of denervation, thereby reducing atrophy and maladaptive voice behaviors [1, 2]. We also aim to preserve neural input through RLN reinnervation whenever possible, and therefore avoid overinjection, instead using standard injection volumes and emphasizing postoperative speech therapy. Prior studies have shown that such an approach can reduce the need for open thyroplasty [1, 2].

Nevertheless, glottic closure continues to change dynamically after denervation. Even with successful early IL, progressive muscular atrophy may widen the glottic gap over time [3]. Although early failure may be related to rapid HA absorption in some patients, this case demonstrated persistent HA prior to the second injection, and the favorable response after supplemental IL suggests that ongoing atrophy and lack of continuous speech therapy were the major contributors to the worsening voice quality [4].

Supplemental IL is safe and effective when indicated. At our institution, fewer than 2% of early‐IL patients require a second injection within 6 months. However, timely supplementation is appropriate if the gap increases. We therefore emphasize patient counseling: the denervated vocal fold is a dynamic structure, early IL provides an initial benefit, but a small proportion of patients may require supplemental treatment.

Speech therapy remains essential throughout the process [5]. Effective rehabilitation reduces maladaptive compensation, improves intrinsic laryngeal muscle efficiency, preserves lamina propria characteristics, and may decrease the need for additional injections [4]. The deterioration observed when this patient discontinued therapy further supports its importance.

## Funding

The study was supported by the grants from National Science and Technology Council, Taiwan (NSTC 113‐2314‐B‐037‐035, NSTC 114‐2314‐B‐037‐074), Kaohsiung Medical University Gangshan Hospital (KMUGH‐113R004, KMUGH‐114R011), and Kaohsiung Medical University, Kaohsiung, Taiwan.

## Ethics Statement

This study was approved by the Institutional Review Board of Kaohsiung Medical University Hospital (KMUHIRB‐E(II)‐20240333).

## Conflicts of Interest

The authors declare no conflicts of interest.

## Data Availability

The data that support the findings of this study are available from the corresponding author upon reasonable request.
